# Multidisciplinary Bioanalytical Approach to Assess the Anti-Aging Properties of Flower Petals—A Promising Sustainable Cosmetic Ingredient

**DOI:** 10.3390/plants14182869

**Published:** 2025-09-15

**Authors:** Đurđa Ivković, Milan Senćanski, Mirjana Novković, Jelena Stojković-Filipović, Jelena Trifković, Petar Ristivojević, Maja Krstić Ristivojević

**Affiliations:** 1 Innovative Centre of the Faculty of Chemistry Ltd., Studentski Trg 12-16, 11158 Belgrade, Serbia; djurdja@chem.bg.ac.rs; 2 Laboratory of Bioinformatics and Computational Chemistry, Institute of Nuclear Sciences Vinca, National Institute of the Republic of Serbia, University of Belgrade, 11001 Belgrade, Serbia; sencanski@vin.bg.ac.rs; 3 Group for Muscle Cellular and Molecular Biology IMGGE, Institute of Molecular Genetics and Genetic Engineering, University of Belgrade, Vojvode Stepe 444a, 11042 Belgrade, Serbia; mirjana.novkovic@imgge.bg.ac.rs; 4 Clinic of Dermatovenereology, University Clinical Center of Serbia, University of Belgrade-Faculty of Medicine, Dr Subotića Starijeg 8, 11000 Belgrade, Serbia; j.stojkovic-filipovic@med.bg.ac.rs; 5 Department of Analytical Chemistry, University of Belgrade-Faculty of Chemistry, Studentski trg 12-16, 11158 Belgrade, Serbia; jvelicko@chem.bg.ac.rs; 6 Department of Biochemistry, University of Belgrade-Faculty of Chemistry, Studentski trg 12-16, 11158 Belgrade, Serbia

**Keywords:** cultivated petals, anti-aging, phenolic profiling, high performance thin layer chromatography (HPTLC), liquid chromatography coupled mass spectrometry (LC-MS), molecular docking, sustainable cosmetics

## Abstract

The increasing demand for natural, safe, and sustainable ingredients is driving innovation in cosmetic science. This study assessed the anti-aging potential of 17 petal extracts using a multidisciplinary bioanalytical approach. In vitro spectrophotometric assays evaluated anti-wrinkle (anti-elastase), anti-pigmentation (anti-tyrosinase), and antioxidant (DPPH, ABTS) activities, while cytotoxicity was tested on HaCaT keratinocytes. Chemical profiling using HPTLC and UHPLC–MS/MS identified 17 phenolic compounds. For the first time, petals from prairie rose (*Rosa setigera Michx.*), common peony (*Paeonia officinalis* L.), horse-chestnut cultivars (*Aesculus hippocastanum* L., *Aesculus × carnea Zeyx.*), lilac (*Syringa vulgaris*), mock-orange (*Philadelphus pubescens Loisel*), orange lily (*Lilium bulbiferum* L.), garden tulip (*Tulipa gesneriana* L.), ivy geranium (*Pelargonium × peltatum* (L.) *L’Hér. ex Aiton*), and wallflower (*Erysimum × cheiri (L.) Crantz*) were studied for their skin anti-aging properties. Prairie rose, peony, and ivy geranium extracts showed strong anti-elastase activity; rose and peony also demonstrated high antioxidant potential, while lilac exhibited significant anti-tyrosinase effects. Key phenolic constituents—caffeic acid, *p*-coumaric acid, and gallic acid—were further examined via molecular docking, which confirmed their inhibitory properties by revealing inhibition mechanisms. All extracts were confirmed to be non-toxic in zebrafish acute toxicity assays at relevant concentrations. This integrative strategy effectively links chemical composition with biological activity, offering valuable insight into the development of safe, plant-derived anti-aging agents for sustainable cosmetic applications.

## 1. Introduction

According to Future Market Insights, the global herbal beauty products market is projected to reach USD 135.9 billion by 2034, driven by growing demand for natural and sustainable skincare solutions. This growth reflects a broader consumer trend toward plant-based ingredients, driven by increasing awareness of the potential long-term risks linked to synthetic formulations [1], highlighting herbal resources as valuable reservoirs of bioactive compounds and reinforcing the need for systematic investigation of underexplored plant species and the development of innovative cosmetic ingredients. However, sustainable harvesting methods are crucial to avoid ecosystem disruption. Prioritizing petals over roots or bark embodies a truly sustainable harvesting approach: it allows plant regeneration, minimizes ecosystem disruption and habitat loss, and aligns with zero-waste and circular-economy principles [2]. This practice not only preserves biodiversity and safeguards long-term resource availability, but also supports local economies through less destructive harvesting, while delivering potent bioactive compounds for eco-friendly cosmetics. Although flowers often exhibit high levels of biological activity and concentration of bioactive compounds, research and industrial applications have traditionally focused more on other plant parts. Extracts from leaves and aerial tissues are predominantly used in cosmetic formulations, while the potential of petals remains largely underexplored [3,4]. Skin aging is a multifactorial process influenced by both external and intrinsic factors. External contributors include ultraviolet (UV) radiation, pollution, and oxidative stress, while intrinsic mechanisms involve genetic predisposition, increased activity of age-related enzymes, and cumulative cellular damage. The visible signs of skin aging—such as fine lines, wrinkles, reduced elasticity, and uneven texture—are primarily attributed to the gradual degradation of collagen and elastin fibers, the decline in hyaluronic acid levels, and compromised integrity of the extracellular matrix (ECM) [5]. Keratinocytes play a key role in protecting the skin against oxidative stress by activating protective signaling pathways, such as the Nrf2/ARE pathway, [6] and producing antioxidant enzymes, such as superoxide dismutase, catalase, and glutathione peroxidase, that neutralize free radicals. These actions help preserve cellular function and maintain the epidermal barrier, thereby slowing the skin aging process. Furthermore, overactivation of tyrosinase—the key enzyme in melanin biosynthesis—is linked to aging-related skin changes, including freckles, wrinkling, and hyperpigmentation [7].

Skin anti-aging evaluation methodology involves a set of parameters, most notably the determination of inhibitory activity against free radical models and age-related enzymes. In vitro enzymatic assays, including anti-collagenase, anti-elastase, anti-hyaluronidase, and tyrosinase inhibition tests, remain crucial for elucidating molecular anti-aging mechanisms [7]. However, these assays are often combined with instrumental techniques to gain insight into the specific compounds responsible for the observed effects. A wide range of analytical techniques has been employed to identify bioactive compounds with targeted efficacy. Advanced chromatographic techniques hyphenated with mass spectrometry and UV/Vis detector spectrometric platforms, such as LC-MS/MS, and UPLC-MS, have become central tools for profiling complex plant extracts and correlating chemical fingerprints with biological activity. For instance, Hypericum species (particularly *H. calycinum*) have been investigated through UPLC-MS and enzyme inhibition assays (collagenase, elastase, hyaluronidase, and tyrosinase), revealing significant anti-aging and skin-whitening properties, largely attributed to their high phenolic content, such as chlorogenic acid [8]. Similarly, UPLC-MS-based untargeted metabolomics has been applied to assess the antioxidant and anti-aging potential of *Rosmarinus officinalis*, where phenols presence significantly correlated with observed potential [9]. NMR-based metabolomics studies, such as those by Hussin et al., have further highlighted *Persicaria minus* as a potent source of anti-aging compounds (such as quercetin, quercetin-3-*O*-rhamnoside, catechin, isorhamnetin, astragalin and apigenin) due to its radical scavenging capacity [10]. In our previous work, we developed a cost-efficient, multi-tiered analytical strategy combining HPTLC chemical profiling, in vitro bioassays (tyrosinase, elastase, DPPH·/ABTS·), and partial least squares (PLS) regression to establish correlations between phenolic markers and bioactivity. To strengthen target validation, molecular docking was employed to confirm enzyme–ligand interactions, offering a high-throughput, mechanism-driven approach for discovering novel skin anti-aging agents [11].

Petals have been investigated to a limited extent in the context of anti-aging, primarily through radical-scavenging assays [12,13]. Their biological activities are largely attributed to phenolic compounds, flavonoids, phenylpropanoids, and secoiridoids. The bioactive properties of rose petals, in particular, are widely recognized, and they are commonly used in cosmetic formulations. Considering the broad spectrum of bioactive compounds detected in the petals studied, they are regarded as promising candidates for further evaluation of anti-aging potential.

Building on these findings, this study establishes a robust multidisciplinary workflow for screening, characterizing, and ensuring the safety of petal extracts, as natural anti-aging agents. The proposed analytical approach enables ed a comprehensive set of experiments. Chemical profiling was performed using both High-Performance Thin-Layer Chromatography (HPTLC) and Ultra-High-Performance Liquid Chromatography-MS/MS (UHPLC-MS/MS). Determination of anti-aging parameters included assessment of radical-scavenging (DPPH^•^, ABTS^•+^), and ECM-degrading and pigmentation-related enzymes inhibition (elastase and tyrosinase, respectively) assays, as well as cytotoxicity assessment (HaCaT cell line), essential for identifying efficient cosmetic ingredients. At 5 µg/mL, most petal extracts (PEs) showed no cytotoxic effect on HaCaT cells, whereas higher concentrations (100–1000 µg/mL) led to a dose-dependent decrease in viability for several extracts, with PE 14 being consistently the most cytotoxic. After screening 17 petal extracts (PEs), toxicity of the four, most efficient petals suitable for cosmetic applications–prairie rose (*Rosa setigera Michx*.), common peony (*Paeonia officinalis* L.), common lilac (*Syringa vulgaris*), and ivy geranium (*Pelargonium × peltatum* (L.) *L’Hér. ex Aiton*)—was determined using in vivo zebrafish (*Danio rerio*) assay. Thus, the main goal of this study was to investigate Serbian flower petals as underexplored and sustainable raw materials and to identify the most potent extracts and their safe concentrations that could be potentially used as effective anti-aging agents in cosmetic formulations. Additionally, molecular docking studies were conducted to elucidate the binding interactions of bioactive compounds with key age-related enzymes, providing insights into their potential mechanisms of action. Notably, several PEs were evaluated for anti-aging activity for the first time, expanding current knowledge and uncovering novel bioactive compounds with significant anti-aging potential and favorable safety profiles for sustainable cosmetic use.

## 2. Results and Discussion

### 2.1. HPTLC Phenolic Profiles of PEs

**Figure 1:**
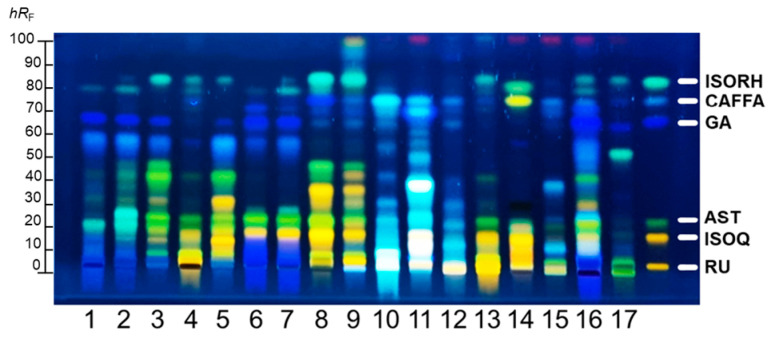
HPTLC profiles of 17 investigated PEs after derivatization with NP reagent: inspection under 366 nm.

The visual examination of the HPTLC chromatograms showed both similarities and variations within the phenolic profiles of the PEs (Figure 1).

Several phenolic compounds, such as rutin (RU, *hR*_F_ 5), isoquercetin (ISOQ, *hR*_F_ 15), astragalin (AST, *hR*_F_ = 23), gallic acid (GA, *hR*_F_ 65), caffeic acid (CAFFA, *hR*_F_ 75), and isorhamnetin (ISORH, *hR*_F_ 83) in the PEs were identified by HPTLC using reference standards (Sigma Aldrich, St. Louis, MO, USA). Almost all PEs exhibited abundant phenolic profiles, with PE5, PE8, PE10, PE11, and PE16 having the most intensive zones. In contrast, PE15 and PE17 exhibited the poorest chemical profiles with zones of the lowest intensity. In addition to PEs of species belonging to the same families (PE6 and PE7; PE8 and PE9), rose PEs formed two distinct clusters based on chemical profile differences: PEs 1–2 and PEs 3–5. Notably, PE10 and PE11, although taxonomically unrelated, exhibited a notable chemical similarity, particularly in the upper part of the chromatogram (>60 *hR*_F_) and in the presence of compounds showing very similar emission spectra (same fluorescence color), likely indicating that they contain the same classes of compounds. RU was primarily detected in PE4, PE8, PE9, PE11, PE13, and PE14. PEs 3–9, PE13, PE14, and PE16 predominantly contained AST and ISOQ, with the most intense bands observed in PE6, PE7 and PE8. PE6, and PE7 were characterized by high levels of GA. GA was also detected in significant amounts in PE1, PE2, PE3 and PE16. PE8 and PE9 showed remarkably similar profiles dominated by RU, ISOQ, AST, ISORH, CAFFA, and ISORH. PE10 and PE11 were distinct, featuring the most intense chromatogram zones and high levels of CAFFA. PE13, though obtained from a different plant family, closely resembled the PE4 profile. CAFFA was also present in smaller amounts in PE9, PE12, PE15, and PE16. Finally, ISORH was detected in PE3, PE8, and PE9 as a high-intensity zone, while in PE4, PE5, PE13, PE16, and PE17 it was detected as a zone of low to moderate intensity. PE17 displayed only a few bands, including GA, CAFFA, and ISORH.

HPTLC enables high-throughput screening of numerous samples with minimal preparation, while providing clear and direct visual differentiation and identification of band intensities. It is especially well-suited for rapid chemo profiling of phenolics in petal extracts, as it provides direct analysis of constituents within the extracts and allows comparative assessment of profiles across multiple samples. The resulting data can be correlated with other techniques, such as UHPLC analysis and bioassays, to rationalize the observed biological activities and guide the selection of the most promising extracts.

### 2.2. UHPLC-DAD-MS/MS

The combined use of HPTLC fingerprinting and UPLC–MS/MS characterization enhances the reliability of our anti-aging evaluation by integrating rapid, visual comparative profiling with high-resolution identification and (semi)quantification of both major and minor PEs bioactive constituents. This approach strengthens the correlation between chemical signatures and the observed skin anti-aging activities. Liquid chromatography coupled with mass spectrometry (LC-MS) revealed the presence of 17 phenolic compounds, including 4 phenolic acids, 6 flavonoids, 6 glycosides, and 1 hydroxycoumarin (Table 1).

The most abundant phenolic in the PEs was GA, ranging from 0.03 ± 0.01 to 50.55 ± 1.79 mg/kg. Similar based on HPTLC results, GA was detected in the highest concentration in PE16 (50.55 ± 1.79 mg/kg) and also significantly in PEs obtained from the Paeoniaceae family plants, such as PE6 and PE7 (46.90 ± 1.20 and 39.71 ± 1.67 mg/kg, respectively), consistent with literature reports and HPTLC profile (Figure 1) highlighting the presence of GA and their derivatives in this family [14]. While most studies focus on Paeoniaceae roots [15], limited research is available on its flowers. Except in peonies, GA was found to be significantly present in roses (PEs 1–5), with concentrations ranging from 2.28 ± 0.09 to 25.77 ± 0.42 mg/kg, as confirmed by HPTLC.

The second most abundant phenolic was AST, detected in the range from 0.14 ± 0.10 to 15.87 ± 1.77 mg/kg, with the highest concentration in PE6 (15.87 ± 1.77 mg/kg). According to available literature, this is the first time that this kaempferol glucoside has been identified in the common peony petals (PE6). However, its occurrence has been previously reported in wild peony (*Paeonia mascula* (L.) Mill.) (PE7), aligning with our results (9.12 ± 0.42 mg/kg), and in the flowers of other Paeoniaceae members [15,16]. It also appeared prominently in nearly identical concentrations in PE14 (15.39 ± 0.92 mg/kg) and PE8 (14.82 ± 1.64mg/kg). AST is reported as a main metabolite of white chestnut (*Aesculus hippocastanum* L.) flowers (PE8), as we confirmed by LC-MS and HPTLC as well [17]. However, there is no report to date about AST presence in wallflower (*Erysimum × cheiri (L.) Crantz*) flower (PE14), although compounds with a similar skeleton, such as kaempferol, are reported [18]. Moderate levels of this flavonoid were found in PE9 (3.50 ± 0.26 mg/kg) and PE16 (4.89 ± 0.12 mg/kg). To the best of our knowledge, this is also the first report of AST detected in ivy geranium (PE16), although the members of the Geraniaceae family contain AST and other kaempferol derivatives [19]. Lower amounts were found in the roses (PE3 (*Rosa abietina Gren.ex Christ*), PE4 (*Rosa × damascena* Mill.), PE5 (*Rosa × damascena Herrm.*)); 1.1 ± 0.1–4.6 ± 0.3 mg/kg). This trend of AST quantification is highly correlated with HPTLC identification, further confirming the detection of the mentioned phenolics (Figure 1).

CA was the most abundant phenolic in mock-orange extract (PE11; 46.73 ± 2.13 mg/kg). Mock-orange (*P. pubescens*, Hydrangeaceae) is a deciduous, frequently cultivated ornamental shrub in gardens across Serbia—alongside its close relative *P.coronarius*—valued for its prolific, fragrant white blossoms. To date, the chemical composition of mock-orange, as well as its floral metabolome, has remained entirely unexplored. Moreover, limited data are available on the Hydrangeaceae species composition, with reports of antimicrobial and anti-inflammatory, hepatoprotective, and antidiabetic properties [20]. The study by Klečáková J. et al. [21] is the only work reporting the presence of phenolics—such as RU, Q, K, ISORH, naringenin, eriodictyol, CAFFA, and ferulic acid—in *P. coronarius* petals. LC-MS analysis revealed that CA is by far the most abundant metabolite in the flowers of this plant, accompanied by significant levels of RU (5.98 ± 0.23 mg/kg), AST (1.97 ± 0.09 mg/kg), HYP (2.10 ± 0.08 mg/kg), and CAFFA (2.56 ± 0.14 mg/kg). These findings provide valuable insight, considering the complete absence of prior phytochemical characterization of this ornamental species. With the exception of PE11, CAFFA was found in significant quantities only in PE10 (2.68 ± 0.12 mg/kg) and PE15 (1.59 ± 0.06 mg/kg).

ISORH was prominent in PE15 (3.77 ± 0.11 mg/kg), PE7 (2.20 ± 0.07 mg/kg), and PE6 (1.86 ± 0.09 mg/kg). ISORH glycosides are detected in marigold (*Calendula officinalis* L.), consistent with our detection of ISORH-3-*O*-R (5.07 ± 0.15 mg/kg) and ISORH-3-*O*-G (1.58 ± 0.05 mg/kg) [22]. Although PE15 showed the highest ISORH content, its HPTLC spot was comparatively weak. This discrepancy likely stems from differences in detection: TLC relies on derivatization and spot intensity, which can be affected by matrix effects or co-migrating compounds, while LC-MS provides more specific and quantitative identification.

RU was found in higher concentrations in PE4 (16.96 ± 1.10 mg/kg), PE8 (14.57 ± 1.23 mg/kg), PE5 (6.50 ± 0.20 mg/kg), PE11 (6 ± 1 65.98 ± 10.23 mg/kg), PE9 (5.60 ± 0.55 mg/kg), and PE13 (3.19 ± 0.26 mg/kg), aligning with HPTLC profiles. Phenolic acid *p*-COUM was detected only in PE10 in higher amounts (1.50 ± 0.02 mg/kg), which is a result consistent with the literature [23]. Other notable compounds included LU (4.08 ± 0.11 mg/kg), K (5.67± 0.93 mg/kg), and EC (13.83 ± 0.56 mg/kg), mainly in PE.

Based on extraction yields (Table S1), Rosaceae and Paeoniaceae petals show higher extractability, reflecting a greater abundance of polar secondary metabolites—particularly phenolics and flavonoids—while Liliaceae and Fabaceae petals yield less, consistent with their lower phenolic contents (Table 1).

Although other phenolics were present in trace amounts, their cumulative effect may contribute to the overall activity of specific extracts. Conversely, potential antagonistic interactions among certain compounds cannot be excluded, which may modulate or reduce the bioactivity in some samples. In conclusion, LC-MS provides highly sensitive, specific, and quantitative profiling of complex phenolic mixtures, enabling precise identification and confirmation of compounds that may be underestimated or overlooked by HPTLC alone.

### 2.3. Skin-Antiaging Assays

#### 2.3.1. Tyrosinase and Elastase Inhibition Assays

To evaluate the efficacy of PEs in inhibiting pigmentation and wrinkle formation, assays targeting tyrosinase and elastase activities were conducted using common spectrophotometric protocols. The tyrosinase (TI) and elastase inhibitory (EI) properties of PEs remain largely unexplored. To the best of our knowledge, this research is the first to examine the tyrosinase, as well as the elastase inhibition potential of flowers from the following species: roses (Rosaceae: prairie rose (*Rosa setigera Michx.*, PE1), purple-yellow rose, (*Rosa odorata (Andrews) Sweet Valentina*™PE2), white rose (*Rosa abietina Gren. ex Christ*, PE3)), peonies (Paeoniaceae: common (*Paeonia officinalis* L., PE6) and wild (*Paeonia mascula* (L.) Mill., PE7), horse-chestnuts (Sapindaceae: white (*Aesculus hippocastanum* L., PE8) and red (*Aesculus* × *carnea Zeyx.*, PE9), common lilac (Oleaceae: *Syringa vulgaris*, PE10), hoary mock-orange (Hydrangeaceae: Philadelphus pubescens Loisel, PE11), garden tulip (Liliaceae: *Tulipa gesneriana* L., PE12), orange lily (Liliaceae: *Lilium bulbiferum* L.PE13), wallflower (Brassicaceae: *Erysimum* × *cheiri* (L.) *Crantz*, PE14), and ivy geranium (Geraniaceae: *Pelargonium* × *peltatum* (L.) *L’Hér. ex Aiton*, PE16).

In our study, most tested PEs exhibited poor to moderate TI, with IC_50_ values ranging from 272 ± 28 µg/mL to 3096 ± 26 µg/mL (Table 2).

PE10 (272 ± 28 µg/mL) demonstrated comparably high TI capacity against standard inhibitor kojic acid (KA) (50 ± 14 µg/mL). Together with PE11 (618 ± 51 µg/mL), they did not differ statistically from KA. Our research showed that the common lilac TI potential is twice as effective as that reported in the only prior study on this plant’s leaves [24]. PE11 (618 ± 51 µg/mL), PE8 (924 ± 4 µg/mL), PE15 (1090 ± 52 µg/mL), PE9 (1192 ± 19 µg/mL), and PE14 (1204 ± 100 µg/mL) exhibited moderate TI, while PE2 (2812 ± 96 µg/mL) and PE17 (Fabaceae: *Robinia pseudoacacia* L., (black locust)) (3096 ± 26 µg/mL) showed low TI. PE6, PE7, PE12, PE13, and PE16 showed negligible, significantly different TI values and were thus considered inactive in the inhibition of this enzyme.

PE11 demonstrated the second-highest tyrosinase inhibitory activity. Although its chemical composition is still largely uncharacterized, related species within the Hydrangeaceae family have exhibited significant inhibitory effects, highlighting the need for further investigation of this and other underexplored taxa within the family [25]. Among all rose species, damask rose (Rosaceae, *Rosa damascena*: PE4 (red) and PE5 (yellow)) has been the most extensively studied and has consistently demonstrated strong tyrosinase inhibitory (TI) activity, as confirmed by various analytical methods. In contrast, our cultivar showed negligible activity based on TLC-DB results, with only a single active zone observed in the PE of the Turkish rose [26]. Conversely, the Thai *R. damascena* water extract exhibited notably higher TI values compared to ours [27]. The other rose PEs, investigated for the first time in this study, also showed low TI activity, with PE2 displaying slightly higher inhibitory potential than the rest.

To date, limited available data on TI pertain primarily to the common peony seeds, where the observed inhibitory activity has been attributed to resveratroloside [28]. In addition, studies have also reported the TI potential of the seeds and roots of *P. mascula* [29,30]. However, the flowers of the investigated peonies did not show TI potential. To the best of our knowledge, this is the first investigation of chestnut extracts (PE8 and PE9), which demonstrated moderate tyrosinase inhibitory (TI) activity (PE8, 924 ± 4 µg/mL; PE9: 1192 ± 19 µg/mL). Although moderate TI activity has previously been reported for *Aesculus hippocastanum*, that finding refers specifically to the fruit extract [31]. Given these preliminary results, further comprehensive studies are needed to fully evaluate its potential. Similarly, only one study to date has investigated the flowers of sweet geranium (PE16)—a species taxonomically related to ivy geranium. While sweet geranium flowers exhibited strong TI activity [32], our PE16 showed no inhibitory effect. The wallflower extract (PE14), also examined for the first time, showed notable TI activity (1204 ± 100 µg/mL), indicating the need for further in-depth investigation. Marigold extract (Asteraceae: *Calendula officinalis* L., PE 15) displayed moderate TI activity, in agreement with existing literature (1.65 ± 0.05 mg/mL) [33].

*p*-COUM, which was quantified in the highest amount in PE10 (and in high levels in PE8), is a confirmed tyrosinase inhibitor [34]. CAFFA, a moderate tyrosinase inhibitor, was most abundant in PE10 and PE11, which also showed the strongest tyrosinase inhibition [33]. Moreover, RU was present in all extracts exhibiting inhibitory activity toward tyrosinase and is indeed reported as an effective inhibitor of this enzyme [35].

Our results revealed that the plant extracts (PEs) exhibited greater elastase inhibitory (EI) activity than tyrosinase inhibitory (TI) activity (Table 2). All PEs demonstrated significantly stronger EI than TI, except for PE10, PE11, and PE16. The IC_50_ values for EI ranged from 206 ± 3 µg/mL to 931 ± 3 µg/mL.

Among the tested extracts, PE16 showed the most potent, statistically significantly different elastase inhibition (206 ± 3 µg/mL), followed by PE1 (222 ± 8 µg/mL) outperforming the standard inhibitor EGCG (348 ± 9 µg/mL). Although PE6 (230 ± 1 µg/mL) showed stronger inhibition than EGCG, the difference was not statistically significant. Extracts from the Paeoniaceae family also demonstrated strong EI activity, with PE6 (230 ± 1 µg/mL) being twice as potent as PE7 (460 ± 22 µg/mL). To date, only one study has examined the aerial parts of Greek *P. mascula*, reporting negligible EI activity, which further highlights the promising EI potential of peony petals [36]. Among the rose extracts, aside from PE1, PE2 (554 ± 54 µg/mL) and PE5 (701 ± 6 µg/mL) also exhibited notable EI activity. This finding is particularly valuable, considering that only *R. damascena* has been extensively studied in this context so far [37].

Additionally, PE8 (931 ± 3 µg/mL) and PE9 (553 ± 1 µg/mL) showed moderate EI potency. Despite their similar TI, PE9 inhibits elastase more effectively than PE8. LC-MS findings suggest a potential correlation between the elevated content of ISORH-3-*O*-R and increased elastase inhibition, as this compound was uniquely present at a significantly higher level in PE9 compared to PE8. Although no direct evidence of EI by this glycoside is reported, it is reported that isorhamnetin’s presence in plants correlates with plant EI [38], warranting further targeted investigation of its glycoside against elastase.

This study is the first to report that flowers of lilac (PE10), mock-orange (PE11), garden tulip (PE12), orange lily (PE13), and wallflower (PE14) have low but significant EI (IC_50_ > 1000 µg/mL), warranting deeper exploration of these plants’ anti-aging potential.

It is possible that part of the elastase inhibitory activity observed in extracts 1, 6, and 16 is attributable to high amounts of AST and GA, which have been reported in the literature as elastase inhibitors [39,40]. LU and K, also present in these extracts, are described as moderate inhibitors, whereas quercetin is considered a potent inhibitor. In addition, ISOQ (detected by the HPTLC) has been reported to exhibit strong elastase inhibitory potential [40]. Therefore, even in low amounts, the combined presence of these compounds may contribute to the overall inhibition observed.

In conclusion, PE1, PE6, and PE16 are promising for elastase-targeted agents, while PE10 shows strong potential for anti-pigmentation. This study highlights these petals’ cosmetic potential, including the use of their combinations. These PEs were obtained from underexplored species, making it a promising novel resource for cosmetics, warranting further exploration of this and other species from Rosaceae, Paeoniaceae, Oleaceae, and Geraniaceae.

#### 2.3.2. Radical Scavenging (RS) Capacity

To further evaluate the anti-aging potential of selected petals, DPPH• and ABTS•^+^ assays—widely used for assessing radical scavenging (RS) capacity—were employed. The DPPH assay confirmed that all PEs possess RS activity, with values ranging from 7.1 ± 0.1 to 695 ± 12 µmol TE/g. PE6 was statistically significantly different, with the highest RS capacity (695 ± 12 µmol TE/g), followed by PE1 (571 ± 28 µmol TE/g) and PE7 (500 ± 29 µmol TE/g). PE7 showed no statistically significant difference than PE1, and PE16 (392 ± 6 µmol TE/g), suggesting similar RS potency. Petals from the Paeoniaceae family demonstrated the strongest RS potential, likely attributable to their high content of gallic acid (GA) and its derivatives, which are well-documented for their potent radical-scavenging effects [41]. Notably, all extracts with elevated GA content (PE1, PE2, PE6, PE7, and PE16) showed high RS capacity. Among rose extracts, PE2 (220 ± 7 µmol TE/g) displayed considerably higher RS activity, second only to PE1. Furthermore, our rose cultivars demonstrated superior RS properties compared to those previously reported in the literature [42]. In contrast, PE10 (95 ± 7 µmol TE/g), PE11 (105 ± 8 µmol TE/g), and PE5 (122 ± 10 µmol TE/g) formed statistically uniform cluster, showing moderate potential. PE8 (197 ± 14 µmol TE/g) showed low to moderate RS activity. Another cluster of moderate to low activity comprised PE13 (21 ± 1 µmol TE/g), PE14 (22 ± 6 µmol TE/g) and PE15 (19.1 ± 0.1 µmol TE/g). The lowest RS capacity, significantly different, was observed in PE12 (7.1 ± 0.1 µmol TE/g) and PE17 (8.2 ± 0.6 µmol TE/g).

A similar trend was observed in the ABTS assay, which yielded slightly higher values for most extracts, ranging from 5.3 ± 0.2 to 850 ± 20 µmol TE/g, further confirming the RS capacity of the tested petals (Table 2). Overall, PE1, PE6, and PE7 consistently exhibited strong RS activity across both assays, highlighting their promising potential for inclusion in cosmetic formulations aimed at mitigating oxidative stress-related skin aging.

#### 2.3.3. Determination of the Total Phenolic Content (TPC) and Total Flavonoid Content (TFC)

Phenolics and flavonoids are key plant compounds contributing to radical scavenging (RS) capacity, primarily due to their hydroxyl groups, which enable them to neutralize reactive oxygen species and mitigate oxidative stress [43]. The correlation coefficients show a strong relationship between TPC results and DPPH (R^2^ = 0.9497) and ABTS (R^2^ = 0.9321) assays, confirming this trend. Flavonoids contribute less to RS capacity, as indicated by the lower correlation between TFC and both DPPH and ABTS results. This pattern is consistent with findings reported in the literature [43].

The total phenolic content (TPC) assay revealed values in the PEs ranging from 1.1 ± 0.1 to 68 ± 2 mg GAE/g. The highest TPC values were observed in PE6 (68 ± 2 mg GAE/g), PE1 (65 ± 3 mg GAE/g), PE7 (58 ± 1 mg GAE/g), and PE16 (44 ± 1 mg GAE/g), which likely explains their strong RS capacity. Moderate phenolic levels were found in PE8 (40 ± 1 mg GAE/g) and PE2 (26 ± 1 mg GAE/g). These findings were consistent with trends in phenolic compound quantities observed for these PE in the UHPLC-MS/MS quantification results. Notably, PE6 exhibited nearly three times higher TPC than previously reported values (26.75 ± 0.65 mg GAE/g) [44]. In contrast, PE12 (1.1 ± 0.1 mg GAE/g), PE15 (3.6 ± 0.1 mg GAE/g), and PE17 (0.7 ± 0.4 mg RUE/g) had the lowest phenolic contents, in line with their sparse phenolic profiles (Figure 1). The only available study on tulip flowers, conducted on five greenhouse-grown cultivars, reported TPC values approximately ten times higher than those of our PE12, likely due to differences in extraction solvent and low-temperature sample preservation [45]. However, even those elevated values do not suggest especially high phenolic content overall, indicating that tulip petals are generally low in phenolics.

TF content showed a slightly different trend than TPC, ranging from 0.7 ± 0.4 to 285 ± 5 mg RUE/g, with the highest in PE7 (285 ± 5 mg RUE/g), followed by PE10 (80 ± 2 mg RUE/g), PE11 (52 ± 4 mg RUE/g), and PE6 (25 ± 2 mg RUE/g). To date, the only report on wild peony measured the TFC in the aerial parts, revealing values approximately 25 times lower than those observed in our study, suggesting a significantly higher concentration of flavonoids in the flowers compared to other plant parts [46]. The least TFC was determined again in PE17 (0.7 ± 0.4 mg GAE/g).

It is important to note that the comparison between flavonoid quantification by UHPLC-DAD-MS/MS and the spectrophotometric TFC assay for PE7, and PE10, highlights a pronounced discrepancy. UHPLC-DAD-MS/MS, which allows precise identification and quantification of individual flavonoid glycosides and aglycones, yielded substantially lower values (PE7: 59.27 mg/kg; PE10: 7.99 mg/kg) compared to the TFC assay (PE7: 285 ± 5 mg RUE/g; PE10: 80 ± 2 mg RUE/g). This divergence can be attributed to non-specific interactions of the AlCl_3_ reagent with other constituents, and minor matrix components, which lead to overestimation in colorimetric measurements. Therefore, UHPLC-DAD-MS/MS provides a more accurate and reliable quantification of the true flavonoid content in the mentioned extracts.

### 2.4. In Silico Docking Assessment

Molecular docking was used to analyze interactions between major phenolic compounds identified in the most potent petals and elastase and tyrosinase enzymes, helping identify potent inhibitors and their anti-aging mechanisms for developing targeted cosmetic formulations.

#### 2.4.1. Tyrosinase Docking

CAFFA and *p*-COUM, identified in the highest quantities in PE10—the most potent tyrosinase inhibitor—were subjected to molecular docking to evaluate their binding affinities to the tyrosinase enzyme. Docking of CAFFA and *p*-COUM into the tyrosinase structure 2Y9X gave conformations with the lowest binding energies −4.14 and −4.38 kcal mol^−1^, respectively (Table 3).

**Figure 2:**
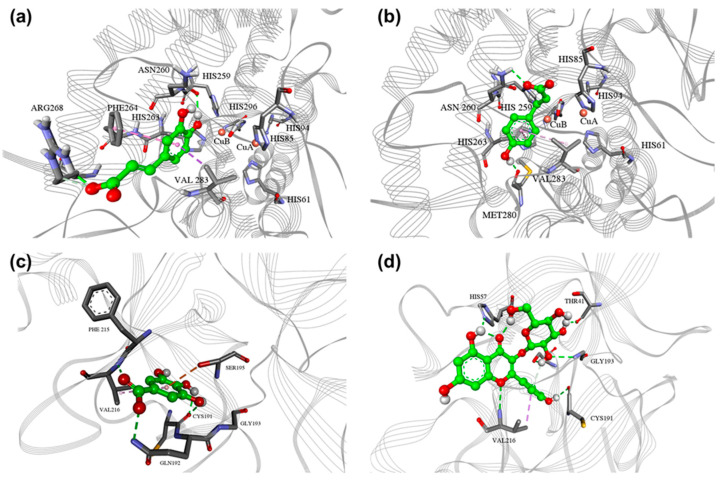
(**a**) CAFFA, (**b**) *p*-COUM, docked into the active site of tyrosinase, and (**c**) GA and (**d**) AST docked into the active site of PPE. The green color represents hydrophilic interactions, and the purple/gray color represents aromatic and other hydrophobic interactions.

Numerous studies have focused on CAFFA docking to tyrosinase, primarily in its oxy form, with fewer studies on modeling met-tyrosinase [47]. Docked conformations of CAFFA and *p*-COUM are near the catalytic site, thus explaining their weak inhibitory profiles. CAFFA forms hydrophilic interactions with amino acid residues Arg268 and Asn260 and hydrophobic interactions with Phe240 and Val283 (Figure 2a). *p*-COUM forms hydrophilic interactions with amino acid residues and hydrophobic interactions with residues (Figure 2b). Although CAFFA and *p*-COUM showed moderate predicted binding affinities toward tyrosinase, their high concentrations suggest they may contribute to the overall inhibitory effect through additive or cumulative mechanisms. Experimentally, *p*-COUM has been reported as a relatively potent human tyrosinase inhibitor (IC_50_ 3 μM), while CAFFA shows weaker inhibition (IC_50_ 250 μM), which is consistent with the trend observed in our docking results (Table 3) [34].

#### 2.4.2. Elastase Docking

Docking of GA—present in significant amounts in PE1, PE6, and PE16, which exhibited the highest elastase inhibitory activity—and AST, the major compound in PE6 and also detected in PEs with moderate EI activity, into the porcine pancreatic elastase (PPE) structure (PDB: 1QGF) resulted in conformations with the lowest binding energies of −4.9 and −6.52 kcal mol^−1^, respectively (Table 3). Some sources imply that GA forms hydrogen bonds with amino acids in the active site of elastase (SER_190, ASN_192, SER_214) in silico experiments [48]. However, our study showed that GA forms hydrophilic interactions with amino acid residues Cys191, Gln192, and Val216, hydrophobic interactions with Val216, and OH-π interaction with Ser195 (Figure 2c), thus explaining the mechanism of inhibition. Our results align with the interaction of GA with Ser195 observed in the literature [49].

Literature indicates that AST does not bind to any amino acid residues on elastase [50]. Only one study has reported a binding energy of −5.65 kcal/mol for AST, suggesting its significant inhibitory properties [51]. Our research, however, has demonstrated slightly higher binding energy and greater inhibitory potency (Table 3). AST forms hydrophilic interactions with amino acid residues Thr41, His57, Cys191, and Gly193 and hydrophobic interactions with Val 216 (Figure 2d). The catalytic triad of elastase consists of His57, Asp102, and Ser195, and these two compounds show interactions with at least one amino acid residue from the list. Therefore, the binding patterns explain their activities as mild EI, with AST showing slightly higher affinity. AST has been reported in the literature as a potent elastase inhibitor (IC_50_ 19.20 ± 3.08 μM), while GA has also demonstrated measurable elastase inhibitory activity [39,40]. These experimental data are in agreement with our docking results, supporting the relevance of these compounds in contributing to the overall elastase inhibition observed in the most active extracts.

It is important to state that this correlation between the presence of GA, AST, CAFFA and *p*-COUM in the active extracts and the observed bioactivity does not imply that other identified phenolic constituents do not contribute to the overall effect; synergistic interactions among different classes of compounds are also possible and should be investigated in future studies, as well as including non-phenolic metabolites.

### 2.5. Toxicity of PEs

#### 2.5.1. Cytotoxic Activity Against HaCaT Cells

**Figure 3:**
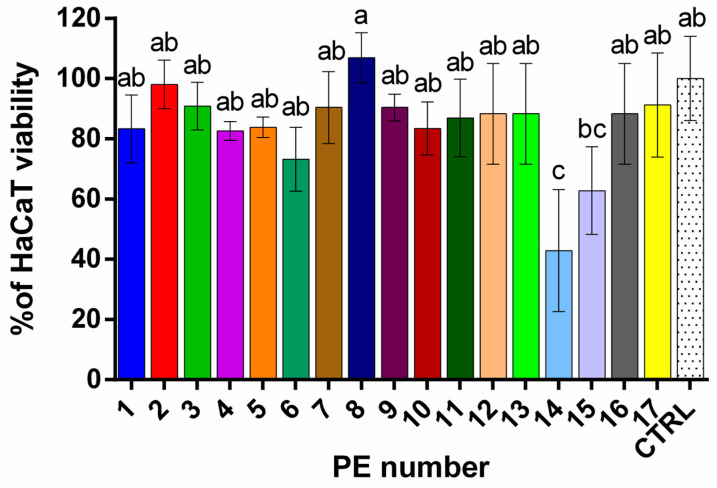
Percentages (%) of HaCaT cell viability after treatment with the investigated PEs (5 µg/mL concentration), as determined by Tukey’s multiple comparison test. Different letters indicate statistically significant differences in cell viability among samples at *p* < 0.05; CTRL stands for untreated control cells.

The cytotoxicity of PEs was determined by monitoring the reduction in viability of immortalized human keratinocytes (HaCaT). A reduction in viability to less than 70% of the control indicates cytotoxic potential [52]. In our study, all PEs were tested at 1000, 500, and 100 µg/mL concentrations (Figure 3 and Figure S1) and were found to be safest at a concentration of 5 μg/mL.

At a concentration of 5 μg/mL, the PEs exhibited differential effects on HaCaT keratinocyte viability, with values ranging from 43 ± 20% to 108 ± 8% (Figure 3). One-way ANOVA revealed a statistically significant overall difference among treatments (*p* = 0.0003, R^2^ = 0.6483). According to Tukey’s multiple comparisons test, PE14 (43 ± 20%) showed a highly significant reduction in viability compared to the control and nearly all other extracts (*p* < 0.05 to *p* < 0.001). Although PE15 (63 ± 15%) demonstrated a viability reduction trend, its effect was not statistically significant compared to the CTRL. PE8 (108 ± 8%) showed the highest viability among the samples, indicating a very slight increase in proliferation that was not statistically significant. All other extracts (PEs 1–13 and 16–17) showed no significant difference from the control (*p* > 0.05) or each other, indicating comparable and non-cytotoxic effects at the tested concentration. Notably, PE14 consistently exhibited significantly reduced viability compared to other extracts. These findings suggest that, except for PE14—and to a lesser extent PE15—the tested extracts did not compromise keratinocyte viability at the lowest concentration tested, supporting their safety for use in cosmetic formulations at this level.

At higher concentrations, the PEs generally exhibited a concentration-dependent decrease in HaCaT cell viability across most samples (Figure S1). Specifically, viability values at 100 µg/mL remained relatively high, indicating mild cytotoxic effects, except for PE9 (6 ± 1%) and PE14 (45 ± 7%). However, at 500 and 1000 µg/mL, a marked reduction in cell viability was observed in several extracts, suggesting increased cytotoxicity at these higher doses. Notably, some PEs maintained moderate viability even at 1000 µg/mL, such as PE1 (80 ± 20%), indicating potential biocompatibility at elevated concentrations.

Viability data indicates a general association with the TPC of the PEs. PEs with high TPC, such as in PE1, PE6, PE7, and PE16, maintain high cell viability at low concentration (5 µg/mL), suggesting the cytoprotective effects of polyphenols. In contrast, PE16, PE8, and PE9 reduce viability at higher concentrations, indicating concentration-dependent cytotoxicity. The data suggests that chestnut petals contain cytotoxic compounds whose mechanisms warrant further investigation and therefore may not be suitable for use in skin formulations at higher concentrations. Prairie rose (PE1) contains non-toxic polyphenols, highlighting its potential for safe cytoprotective applications.

Overall, selecting safe dosages is crucial in potential cosmetic applications. Cytotoxicity testing on HaCaT cells ensures that active concentrations of extracts showing EI, TI, and RS effects are non-toxic to skin cells. This complements the enzyme and antioxidant assays by confirming both efficacy and safety, which is crucial for cosmetic applications.

#### 2.5.2. Developmental Toxicity of Evaluated PEs in Zebrafish Embryos

**Figure 4:**
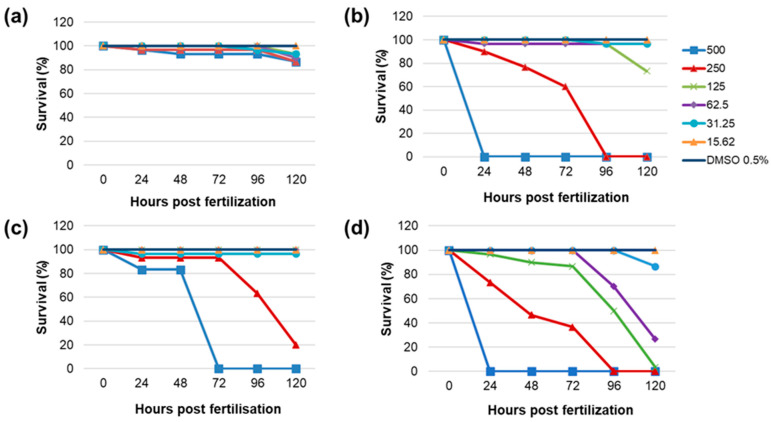
Acute toxicity of different concentrations of tested PEs in zebrafish embryos through 24–120 hpf: (**a**) PE 1; (**b**) PE 6; (**c**) PE 10; (**d**) PE 16.

Four of the most promising PEs were selected for in vivo toxicity determination based on the EI (PE1, PE6, PE16), the TI assay (PE10), and the RS capacity assay (PE1, PE6). All these extracts exhibited low or no cytotoxicity on HaCaT cells, suggesting that the selected PEs are safe in vitro. Toxicity was assessed through the survival and hatchability of zebrafish embryos incubated with six different concentrations of the investigated PEs, ranging from 15.62 to 500 μg/mL, spaced by a constant factor of 2, for up to 120 hpf. PE1 exhibited significant lethality only at 500 μg/mL at 120 hpf (Figure 4a), although the survival rate was 80%, which is notably higher compared to other plant extracts at the same concentration that caused 100% mortality; PE6 caused 100% mortality at 500 and 250 μg/mL (Figure 4b). Further, PE10 led to complete lethality at 500 μg/mL and significant lethality at 250 μg/mL (Figure 4c). As shown in Figure 4d, PE16 resulted in 100% lethality at 500, 250, and 125 μg/mL, with significant lethality observed at 62.5 μg/mL.

**Figure 5:**
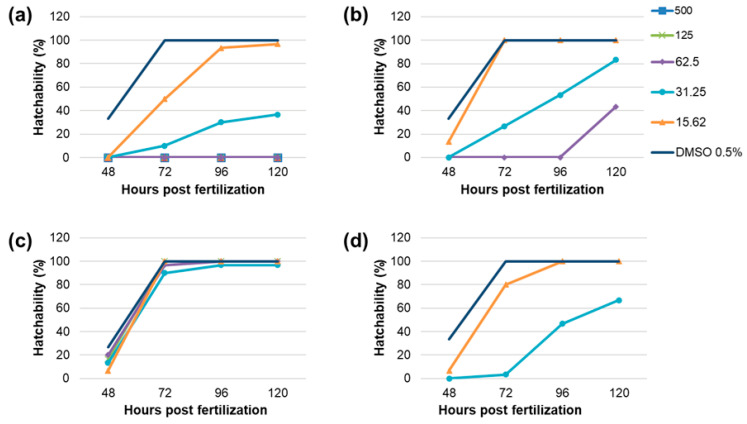
Influence of different concentrations of tested PEs on hatchability of zebrafish embryos through 48–120 hpf: (**a**) PE 1; (**b**) PE 6; (**c**) PE 10; (**d**) PE 16.

The lowest LC_50_ was observed for PE16 at 48.13 μg/mL, followed by PE10 at 220.85 μg/mL, and PE6 at 226.12 μg/mL. The LC_50_ for PE1 could not be determined, as the highest concentration tested caused only 20% lethality. Concentrations causing lethality ≥80% have not been considered for testing hatching inhibition. PE1 caused complete hatching inhibition at concentrations ≥62.5 μg/mL by 120 hpf (Figure 5a). Hatching inhibition was also observed at 31.25 μg/mL, but 15.62 μg/mL did not affect hatching by 120 hpf. Significant hatching inhibition was observed with PE6 extracts at 125 and 62.5 μg/mL, whereas lower concentrations (31.25 and 15.62 μg/mL) did not affect hatchability by 120 hpf (Figure 5b). PE10 extracts showed no significant hatching inhibition at concentrations ≤125 μg/mL (Figure 5c). PE16 affected hatchability at concentrations of 31.25 μg/mL (Figure 5d), while 15.62 μg/mL did not impact hatchability.

Taken together, in vivo toxicity evaluation confirmed the safety of PE6, PE10, and PE16 at ≤31.25 μg/mL, and PE1 at ≤15.62 μg/mL. These findings are in agreement with the MTT assay results, where the same extracts exhibited no cytotoxic effects on HaCaT cells at 5 μg/mL. Interestingly, although PE1 caused only 20% lethality at the highest tested concentration, it significantly inhibited hatching even at lower concentrations (≥31.25 μg/mL). This suggests that PE1 may not exert acute toxicity, but could interfere with normal embryonic development, pointing to a possible mechanism of developmental toxicity. In contrast, PE16 demonstrated the highest acute toxicity, with 100% mortality at concentrations ≥125 μg/mL. These results highlight the importance of assessing both viability and developmental endpoints to ensure the safety of PEs for potential cosmetic applications. Zebrafish assays assess whole-organism effects, including bioavailability and toxicity, complementing in vitro enzyme and antioxidant tests. This provides a whole picture of the extracts’ anti-aging potential.

## 3. Materials and Methods

### 3.1. Reagents and Chemicals

Kojic acid (≥98.5%, KA), epigallocatechin gallate (EGCG), gallic acid ((≥98%, GA), caffeic acid (HPLC purity, CAFFA), chlorogenic acid (≥95%, CA), rutin hydrate (HPLC purity, RU), quercetin (≥95%, Q), quercetin-3-*O*-glucoside (≥98%, ISOQ), kaempferol-3-*O*-glucoside (≥95%, AST), kaempferol (≥97%, K), (-)-epicatechin (≥98% from green tea, EC), aesculetin (≥98%, AES), *p*-coumaric acid (≥98%, *p*-COUM), hyperoside (≥95%, HYP), naringin (≥95%, NAR), isorhametin-3-*O*-rutinoside (≥95%, ISORH-3-*O*-R), isorhametin-3-*O*-glucoside (≥98%, ISORH-3-*O*-G), quercetin-3-*O*-rhamnoside (≥95%, Q-3-*O*-R), isorhamnetin (≥95%, ISORH), luteolin ((≥98%, LU), 3,4-dihydroxy-l-phenylalanine (L-DOPA), tyrosinase from mushroom, porcine pancreatic elastase (PPE), N-succinyl-Ala-Ala-Ala-*p*-nitroanilide (≥98%), 2,2′-Azino-bis(3-ethylbenzothiazoline-6-sulfonic acid) diammonium salt (ABTS), 6-hydroxy-2,5,7,8- tetramethylchroman-2-carboxylic (TROLOX), 2-aminoethyl diphenylborinate (Natural Product reagent), tricin, dimethyl sulfoxide (DMSO), sodium-chloride, magnesium chloride, calcium chloride, and polyethylene glycol (PEG) were purchased from Sigma Aldrich (St. Louis, MO, USA). 2,2-Diphenyl-1-picrylhydrazyl radical (DPPH) was bought from Fluka (Buchs, Switzerland). Toluene, sodium hydroxide, and methanol (LC–MS purity) were procured from Zorka Pharma (Šabac, Serbia), ethyl acetate from BETAHEM (Belgrade, Serbia), and formic acid from LACH-NER (Neratovice, Czech Republic). Sodium carbonate, potassium persulfate, sodium dihydrogen phosphate, sodium hydrogen phosphate, and Folin–Ciocalteu (FC) reagent were obtained from Merck (Darmstadt, Germany). Aluminum chloride and sodium nitrite were bought from Centrohem (Stara Pazova, Serbia). Dulbecco’s Modified Eagle’s Medium (DMEM) powder, non-essential amino acids (NEAA, 100X), and antibiotic-antimycotic solution (100X) were obtained from Gibco (Paisley, UK). Fetal bovine serum (FBS), L-glutamine, and sodium pyruvate were sourced from Biological Industries (Cromwell, CT, USA).

### 3.2. Petal Material and Preparation of Extracts

#### 3.2.1. Petal Sampling

Petals from 17 plants (roses cultivars (PE1, PE2, PE3, PE4, PE5), peonies (PE6, PE7), white horse-chestnuts (PE8), common lilac (PE10), hoary mock-orange (PE11), garden tulip (PE12), orange lily (PE 13), wallflower (PE14), ivy geranium (PE16), and black locust (PE17) were cultivated in Serbia (Lazarevac, Serbia) and collected during May and June 2022 primarily from private gardens. Flowers of red horse-chestnuts (PE9) were gathered at the town park (Belgrade, Serbia), while marigold was bought from Josif Pančić Pharmacy (Belgrade, Serbia). The petals were dissected from plants, sun-dried, and ground using a household blender (Gorenje, Velenje, Slovenia). Voucher specimens were identified by Peđa Janaćković and deposited at the Herbarium of the Department of Biology, University of Belgrade (BEOU), under accession numbers BEOU KMSB 95287–95320. Detailed information about the plants investigated is provided in Table S1.

#### 3.2.2. Petal Material Extraction

Compounds from powdered petals were extracted with methanol using ultrasound-assisted extraction (ELTA 90 Medical Science, Belgrade, Serbia) for 30 min at room temperature (1/10, *m*/*v*). After solvent evaporation on a rotatory evaporator (IKA RV 05, IKA Werke, Staufen, Germany), dried residues were dissolved in methanol to obtain a concentration of 100 mg/mL of petal extracts (PEs). Extraction yields are given in Table S1.

### 3.3. HPTLC Analysis

#### Derivatization with Natural Product Reagent (NPR)

Samples (2 µL of 50 mg/mL PEs and 2.5 µL of 0.17 mg/mL phenolic standards) were applied as 6 mm bands onto silica gel 60 F_254_ plates (Merck, Darmstadt, Germany) using a Linomat 5 applicator (CAMAG, Muttenz, Switzerland). Chromatographic separation was performed with ethyl acetate/toluene/formic acid (10:8:3, *v*/*v*/*v*) after 25 min of chamber saturation, with a solvent front of 70 mm. Plates were derivatized in 0.5% (*w*/*v*) NP reagent, dried, and then immersed in 5% methanolic PEG 400 (*w*/*v*). After final drying, the fluorescent zones were visualized at 366 nm and documented with a mobile phone (Samsung Galaxy S21, Samsung Electronics, Suwon, Republic of Korea) [53].

### 3.4. UHPLC-DAD-MS

We quantified phenolic compounds in PEs using a Dionex Ultimate 3000 UHPLC system equipped with a diode array detector (DAD) and a triple quadrupole mass spectrometer (TSQ Quantum Access Max) (Sunnyvale, CA, USA). Phenolic compounds were determined and quantified using the method previously described and detailed in Protocol S1. The calibration parameters of the phenolic standards are presented in Table S2 [54].

### 3.5. Spectrophotometric Assays

RS capacity assays, TP, and TF content were conducted according to established protocols [54] using a GBC UV-visible Cintra 6 spectrophotometer (Dandenong, VIC, Australia). Anti-tyrosinase assay [54], anti-elastase assay [11], and cytotoxicity assessment [54] were performed using a BioTek 800 TS spectrophotometer (Agilent Technologies, Inc., Headquarters, Santa Clara, CA, USA). Measurements were performed in triplicate for each sample.

#### 3.5.1. Radical Scavenging Capacity Assays

##### (DPPH^●^ Radical Scavenging Assay)

0.1 mL of PE was mixed with 3.9 mL of a 71 µmol/L methanolic DPPH· solution (*w*/*v*). After a 60 min incubation in the dark, absorbance was measured at 517 nm. Results were calculated using a Trolox calibration curve and expressed as micromoles of Trolox equivalents (TE) per gram of initial plant material (µmol TE/g).

##### (ABTS^●+^ Radical Scavenging Assay)

The ABTS^●+^ solution was prepared by mixing equal volumes of a 7 mmol/L ABTS solution and a 2.45 mmol/L potassium persulfate solution, followed by a 17 h incubation. The solution was then diluted with methanol to achieve an absorbance of approximately 0.9 at 734 nm. For analysis, 0.15 mL of PE was added to 2.85 mL of the ABTS^●+^ solution, and absorbance was measured at 734 nm. Results were expressed as micromoles of Trolox equivalents per gram of initial plant material (µmol TE/g).

#### 3.5.2. Total Phenolic Content (TPC) and Total Flavonoid Content (TFC)

For the TPC test, 0.5 mL of each PE was mixed with 2.5 mL of 10% Folin–Ciocalteu’s reagent (*v*/*v*). After 5 min, 2 mL of 7.5% sodium carbonate (*w*/*v*) and 0.5 mL of distilled water were added. Following a 60 min incubation, absorbance was measured at 765 nm. Results were expressed as milligrams of GA equivalents (GAE) per gram of initial plant material (mg GAE/g).

For the TFC test, 0.3 mL of PE was combined with 3.4 mL of 30% methanol (*v*/*v*), 0.15 mL of sodium nitrite (0.5 mol/L), and 0.15 mL of aluminum chloride (0.15 mol/L). After a 5 min incubation, 1 mL of 1 mol/L sodium hydroxide was added, and absorbance was measured at 506 nm. Results were expressed as milligrams of rutin equivalents (RUE) per gram of initial plant material (mg RUE/g).

#### 3.5.3. Inhibition of Tyrosinase (Anti-Tyrosinase Assay)

The anti-tyrosinase activity was assessed using a 96-well microtiter plate method. L-DOPA served as the substrate. Each well contained 140 µL of phosphate buffer (20 mmol/L, pH 6.8), 20 µL of diluted PE, and 20 µL of mushroom tyrosinase (550 IU/mL). The mixture was incubated at 37 °C for 20 min before 20 µL of L-DOPA (5 mmol/L) was added to initiate the reaction. Absorbance was measured at 475 nm after 15 min. Results were expressed as IC_50_ values (µg/mL) and calculated using:

$$I\left(\%\right)=100\times \frac{\left[\left(A-B\right)-\left(C-D\right)\right]}{\left(A-B\right)}$$

where

***A*** = Absorbance of the enzyme with the substrate (positive control)
***B*** = Absorbance of the blank (substrate without enzyme)
***C*** = Absorbance of the sample with the enzyme and substrate
***D*** = Absorbance of the sample blank (sample without enzyme)

KA served as a standard inhibitor in this assay.

#### 3.5.4. Inhibition of Elastase (Anti-Elastase Assay)

The assay quantified *p*-nitroanilide released during the enzyme-substrate reaction at 410 nm. PPE and N-Succinyl-Ala-Ala-Ala-*p*-nitroanilide were prepared in 0.1 mol/L tricine buffer (pH 8). In a 96-well plate, 20 µL of PE, 20 µL of elastase, and 140 µL of buffer were incubated for 40 min. The substrate was then added, and absorbance was monitored after 15 min. EGCG was used as a standard inhibitor. Elastase inhibition was calculated as:

$$I\left(\%\right)=100\times \frac{\left(A-B\right)}{A}$$

where

***A*** = Absorbance of the positive control (enzyme with substrate)
***B*** = Absorbance of the sample (enzyme with substrate and sample)

### 3.6. Docking Studies

The Agaricus bisporus tyrosinase crystal structure in complex with tropolone was downloaded from the RCSB database, PDB code 2Y9X [55]. An oxygen atom was added to the bi-copper active site, as per the crystal structure of 1BT3 [56], as used in earlier studies [57], to meet the structural conditions of the met-state. The crystal structure of the PPE was downloaded from the RCSB Protein Data Bank, PDB code 1QGF [58]. Heteroatoms were removed, and the protein structure was prepared in AutoDock Tools 1.5.6. [59] and initially geometrically optimized using the MMFF94 force field [60]. Ligands were built and optimized using Avogadro 1.2.0 and MOPAC 2016, respectively [61]. Protein and ligand structures were prepared in Autodock Tools 1.5.6 [62]. Grid boxes of 60 × 60 × 60 were centered on the binding site with a spacing of 0.375 Å. Atom charges in the active site were also calculated on the PM7 level of theory. Atom charges were calculated using PM7 theory. Docking simulations were performed using AutoDock GPU with 100 Lamarckian genetic algorithm (LGA) runs [63].

### 3.7. Evaluation of PEs’ Toxicity

#### 3.7.1. HaCaT Viability Assay

Medium (DMEM; Gibco, Paisley, UK) supplemented with 10% fetal bovine serum (FBS), 2 mM L-glutamine, antibiotics (penicillin and streptomycin), nonessential amino acids, and sodium pyruvate. Cells were maintained at 37 °C in a humidified atmosphere containing 5% CO_2_.

For the viability assay, cells were seeded in 96-well plates at a density of 10,000 cells per well and allowed to adhere for 24 h. Subsequently, cells were treated with PEs, while control wells received culture medium only. After 24 h of treatment, cell viability was evaluated using the MTT assay. Briefly, 20 μL of MTT solution (5 mg/mL) was added to each well and incubated for 2 h. The resulting formazan crystals were dissolved in 200 μL of dimethyl sulfoxide (DMSO), and absorbance was measured at 570 nm with a reference wavelength of 630 nm using a microplate reader (BioTek 800 TS spectrophotometer, Agilent Technologies, Inc., Headquarters, Santa Clara, CA, USA).

#### 3.7.2. In Vivo Toxicity of PEs

All experiments involving zebrafish were performed under standard conditions by the EU Directive 2010/63/EU and the ethical guidelines of the Guide for Care and Use of Laboratory Animals of the Institute of Molecular Genetics and Genetic Engineering, University of Belgrade. Zebrafish were maintained in standard conditions at 26 ± 1 °C under a photoperiod of 14 h:10 h light:dark, and fed with brine shrimp, SDS300, and tropical flakes four times a day. Embryos were produced by wild-type adults AB-strain breeding groups. Fertilized eggs were collected within 30 min, rinsed with embryo culture medium (5 mM NaCl, 0.17 mM KCl, 0.33 mM CaCl_2_, and 0.4 mM MgCl_2_, 0.1% methylene blue, pH 7.2—E3), and maintained in a Petri dish at 28.0 °C for treatment. To avoid genetic bias, eggs were collected from a minimum of three breeding groups and mixed.

FET test was performed according to the OECD 2013 guidelines for the testing of chemicals [64]. The embryos were examined under a biological stereomicroscope (Stemi 508 trino with AxioCam 305, Carl Zeiss Microscopy, LLC, Thornwood, NY, USA) at 6 h post-fertilization (hpf). Well-developed healthy embryos were randomly distributed into 24-well plates, each well containing 10 embryos in 1 mL embryo culture medium (E3). Embryos were treated with six concentrations of each plant extract, 15.62, 31.25, 62.5, 125, 250, and 500 μg/mL for 120 hpf and 0.5% DMSO (vehicle) and maintained at 28.0 ± 0.5 °C. E3 water was used as a negative control. Toxicity was assessed through survival and hatchability of embryos at five time points (24, 48, 72, 96, and 120 hpf) compared to the vehicle. The exposure solution was replaced every 24 h. The number of dead individuals and the state of embryonic development were examined daily, and the dead embryos were removed. Tests were performed in triplicate.

### 3.8. Statistical Analysis

All spectrophotometric (DPPH, ABTS, TPC, TFC, TI, EI) as well as HaCaT viability results are expressed as mean ± standard deviation (SD). For following assays: DPPH, ABTS, TPC, TFC, and HaCaT, differences among extracts were analyzed by one-way ANOVA followed by Tukey’s multiple comparison test (α = 0.05). This parametric approach was chosen because the results exhibited low variability and did not deviate markedly from normal distribution, allowing reliable mean-based comparisons.

For enzyme inhibition assays (TI and EI), the data did not meet the assumption of normal distribution, primarily because several PEs did not exhibit measurable IC_50_ values (classified as inactive) and due to the high variability observed across means. This justified the application of non-parametric statistical methods for subsequent analyses. Therefore, a non-parametric approach was applied: the Kruskal–Wallis test followed by Dunn’s multiple comparison test (α = 0.05), which enables ranking-based comparisons across all extracts. Extracts without detectable inhibition (/) were assigned the highest tested IC_50_ value (4000 µg/mL for tyrosinase and 1000 µg/mL for elastase), to avoid misinterpretation that “0” corresponds to maximal activity.

## 4. Conclusions

This study established a comprehensive approach—combining in vitro, in vivo, and in silico methods—for evaluating the anti-aging potential of 17 flower petal extracts cultivated in Serbia. Many of the taxa investigated were analyzed for the first time. Several extracts, particularly those from prairie rose and common peony, demonstrating strong antioxidant and anti-elastase activity, with ivy geranium showing significant anti-elastase activity, and common lilac showing notable anti-tyrosinase potential, represent particularly suitable candidates for development in anti-aging cosmetic formulations. HPTLC and UHPLC-MS/MS revealed diverse phenolic profiles, and molecular docking confirmed the enzyme-binding potential of key quantified compounds. Caffeic and *p*-coumaric acids were identified as weak tyrosinase inhibitors, whereas astragalin and gallic acid moderately inhibited elastase in silico. Toxicity testing on HaCaT cells and zebrafish embryos confirmed good biosafety for most extracts at lower concentrations. Overall, Serbian flower petals represent promising and safe candidates for incorporation into natural anti-aging cosmetic formulations. The complementarity of chromatographic profiling, enzyme bioassays, molecular docking, and toxicity testing provided a multi-dimensional assessment of both efficacy and safety.

## Figures and Tables

**Table 1:** List of quantified phenolic compounds in 17 investigated PEs expressed in milligrams of phenolics per kilogram of petal material (mg/kg) as mean value ± standard deviation.

Phenolics	Phenolic Acids				HQ	Flavonoid Glycosides							Flavonoids				
PEs	GA	CA	*p*-COUM	CAFFA	AES	RU	AST	HYP	NAR	ISORH-3-*O*-R	ISORH-3-*O*-G	Q-3-*O*-R	EC	Q	LU	K	ISORH
1	25.77 ± 0.42	0.04 ± 0.01	0.20 ± 0.02	0.02 ± 0.01	ND	0.81 ± 0.01	1.06 ± 0.01	0.26 ± 0.01	ND	ND	ND	0.58 ± 0.06	ND	0.02 ± 0.01	0.22 ± 0.01	0.05 ± 0.01	0.06 ± 0.01
2	16.46 ± 0.48	0.15 ± 0.03	0.17 ± 0.02	0.02 ± 0.01	ND	0.70 ± 0.10	2.05 ± 0.11	1.36 ± 0.11	ND	ND	0.02 ± 0.01	0.46 ± 0.04	3.19 ± 0.11	0.13 ± 0.01	0.55 ± 0.08	0.74 ± 0.02	0.28 ± 0.02
3	8.39 ± 0.25	0.15 ± 0.03	0.04 ± 0.01	0.02 ± 0.01	ND	0.53 ± 0.03	4.55 ± 0.27	0.37 ± 0.04	0.08 ± 0.01	ND	ND	0.50 ± 0.04	0.05 ± 0.01	0.12 ± 0.01	1.80 ± 0.05	2.16 ± 0.20	0.14 ± 0.01
4	2.28 ± 0.09	ND	0.15 ± 0.02	0.03 ± 0.01	ND	16.96 ± 1.10	4.20 ± 0.26	0.33 ± 0.04	0.70 ± 0.05	0.26 ± 0.02	0.39 ± 0.03	0.26 ± 0.02	5.58 ± 0.21	0.41 ± 0.02	1.46 ± 0.05	1.28 ± 0.09	0.14 ± 0.01
5	7.35 ± 0.21	0.05 ± 0.01	ND	ND	ND	6.50 ± 0.20	4.35 ± 0.28	1.28 ± 0.18	ND	ND	0.01 ± 0.01	2.09 ± 0.05	1.85 ± 0.18	0.57 ± 0.02	1.58 ± 0.05	2.00 ± 0.18	0.22 ± 0.02
6	46.90 ± 1.20	0.07 ± 0.01	ND	0.03 ± 0.01	ND	0.15 ± 0.01	15.87 ± 1.77	2.00 ± 0.05	ND	0.02 ± 0.01	0.76 ± 0.09	0.07 ± 0.01	0.25 ± 0.02	0.08 ± 0.01	1.39 ± 0.06	1.76 ± 0.09	1.86 ± 0.09
7	39.71 ± 1.67	0.02 ± 0.01	0.13 ± 0.01	ND	0.07 ± 0.01	0.09 ± 0.01	9.12 ± 0.42	3.31 ± 0.25	ND	ND	1.29 ± 0.08	0.03 ± 0.01	ND	0.12 ± 0.01	1.28 ± 0.107	1.90 ± 0.05	2.20 ± 0.07
8	2.04 ± 0.05	0.05 ± 0.01	1.34 ± 0.15	0.16 ± 0.02	0.50 ± 0.04	14.57 ± 1.23	14.82 ± 1.64	3.29 ± 0.25	ND	0.12 ± 0.01	0.44 ± 0.05	0.81 ± 0.02	13.83 ± 0.56	1.26 ± 0.03	4.08 ± 0.11	5.67 ± 0.93	0.45 ± 0.05
9	0.03 ± 0.01	0.06 ± 0.01	0.25 ± 0.02	0.02 ± 0.01	0.30 ± 0.04	5.60 ± 0.55	3.50 ± 0.26	0.77 ± 0.09	0.05 ± 0.01	0.52 ± 0.04	0.51 ± 0.05	0.41 ± 0.04	2.11 ± 0.06	0.23 ± 0.02	1.20 ± 0.03	1.47 ± 0.16	0.39 ± 0.07
10	0.42 ± 0.09	0.13 ± 0.03	1.50 ± 0.02	2.68 ± 0.12	0.20 ± 0.03	1.93 ± 0.12	0.14 ± 0.10	0.11 ± 0.01	ND	0.53 ± 0.04	0.03 ± 0.01	ND	ND	0.08 ± 0.01	0.10 ± 0.01	0.06 ± 0.02	0.09 ± 0.02
11	0.44 ± 0.09	46.73 ± 2.13	0.14 ± 0.02	2.56 ± 0.14	0.41 ± 0.02	5.98 ± 0.23	1.97 ± 0.09	2.10 ± 0.08	ND	0.02 ± 0.01	0.01 ± 0.01	ND	ND	0.25 ± 0.02	0.14 ± 0.01	0.10 ± 0.01	0.05 ± 0.01
12	ND	1.71 ± 0.16	ND	0.27 ± 0.01	0.22 ± 0.01	1.29 ± 0.02	0.79 ± 0.08	0.19 ± 0.01	ND	0.19 ± 0.02	0.29 ± 0.02	0.03 ± 0.01	1.00 ± 0.05	0.15 ± 0.02	0.09 ± 0.02	0.03 ± 0.01	0.33 ± 0.04
13	0.03 ± 0.01	ND	ND	0.01 ± 0.01	0.02 ± 0.01	3.19 ± 0.26	0.77 ± 0.09	0.09 ± 0.01	0.10 ± 0.01	0.07 ± 0.01	0.12 ± 0.01	0.06 ± 0.01	1.10 ± 0.05	0.13 ± 0.02	0.17 ± 0.02	0.30 ± 0.02	0.06 ± 0.01
14	0.05 ± 0.01	0.03 ± 0.01	0.29 ± 0.02	0.07 ± 0.01	0.05 ± 0.01	1.29 ± 0.11	15.39 ± 0.92	0.76 ± 0.08	0.07 ± 0.01	0.02 ± 0.01	0.03 ± 0.01	ND	0.05 ± 0.01	0.12 ± 0.02	0.43 ± 0.02	0.20 ± 0.02	0.14 ± 0.01
15	0.07 ± 0.01	4.68 ± 0.34	0.19 ± 0.02	1.59 ± 0.06	0.15 ± 0.02	1.61 ± 0.09	0.30 ± 0.04	0.48 ± 0.07	ND	5.07 ± 0.15	1.58 ± 0.05	ND	0.48 ± 0.03	0.06 ± 0.01	0.08 ± 0.02	0.04 ± 0.01	3.77 ± 0.11
16	50.55 ± 1.79	0.06 ± 0.01	ND	0.70 ± 0.05	ND	2.75 ± 0.21	4.89 ± 0.12	0.62 ± 0.10	ND	0.01 ± 0.01	0.01 ± 0.01	0.28 ± 0.02	1.90 ± 0.01	0.13 ± 0.01	0.90 ± 0.09	1.09 ± 0.05	0.09 ± 0.01
17	0.07 ± 0.01	ND	0.46 ± 0.05	0.04 ± 0.01	ND	0.81 ± 0.05	1.20 ± 0.07	0.08 ± 0.01	0.06 ± 0.01	0.09 ± 0.01	0.05 ± 0.01	0.09 ± 0.01	3.59 ± 0.15	0.06 ± 0.01	0.69 ± 0.04	1.09 ± 0.04	0.20 ± 0.02

ND refers to phenolics that are not detected in the corresponding PEs. HQ stands for hydroxycoumarin group.

**Table 2:** Results of antioxidative capacity (DPPH, ABTS), bioactive content (TPC, TFC), and enzyme inhibition (tyrosinase (TI), and elastase (EI)) assays of PEs expressed as means with standard deviation.

	Antioxidative Capacity		Bioactive Content		IC_50_ Values	
PEs	DPPH (μmol TE/g)	ABTS (μmol TE/g)	TPC (mg GAE/g)	TFC (mg RUE/g)	TI (µg/mL)	EI (µg/mL)
1	571 ± 28 ^b^	577 ± 13 ^bc^	65 ± 3 ^ab^	2.1 ± 0.7 ^e^	>4000 ^g^	222 ± 8 ^a^
2	220 ± 7 ^d^	213 ± 7 ^de^	26 ± 1 ^d^	18 ± 1 ^bc^	2812 ± 96 ^d^	554 ± 54 ^cd^
3	49 ± 3 ^g^	49 ± 1 ^ghi^	7.7 ± 0.1 ^fg^	3.6 ± 1.1 ^de^	>4000 ^g^	>1000 ^f^
4	36 ± 3 ^gh^	38 ± 1 ^hi^	12 ± 1 ^ef^	3.4 ± 0.4 ^de^	>4000 ^g^	>1000 ^f^
5	122 ± 10 ^ef^	146 ± 8 ^ef^	18 ± 1 ^de^	5.8 ± 0.5 ^d^	>4000 ^g^	701 ± 6 ^de^
6	695 ± 12 ^a^	850 ± 20 ^a^	68 ± 2 ^a^	25 ± 2 ^b^	/ ^g^	230 ± 1 ^ab^
7	500 ± 29 ^bc^	640 ± 20 ^ab^	58 ± 1 ^bc^	285 ± 5 ^a^	/ ^g^	460 ± 22 ^bc^
8	197 ± 14 ^de^	246 ± 1 ^d^	40 ± 1 ^cd^	21 ± 6 ^b^	924 ± 4 ^b^	931 ± 3 ^e^
9	28 ± 2 ^hi^	21 ± 1 ^i^	4.4 ± 0.1 ^g^	5.9 ± 0.6 ^d^	1192 ± 19 ^bc^	553 ± 1 ^cd^
10	95 ± 7 ^ef^	97 ± 3 ^fgh^	21 ± 1 ^de^	80 ± 2 ^ab^	272 ± 28 ^a^	>1000 ^f^
11	105 ± 8 ^ef^	88 ± 3 ^gh^	20 ± 1 ^de^	52 ± 4 ^ab^	618 ± 51 ^ab^	>1000 ^f^
12	7.1 ± 0.1 ^i^	5.3 ± 0.2 ^k^	1.1 ± 0.1 ^h^	1.5 ± 0.1 ^f^	/ ^g^	>1000 ^f^
13	21 ± 1 ^hi^	11 ± 2 ^j^	12 ± 1 ^ef^	14 ± 1 ^c^	/ ^g^	/ ^f^
14	22 ± 6 ^hi^	32 ± 2 ^hi^	8.9 ± 0.2 ^f^	6.7 ± 0.1 ^d^	1204 ± 100 ^cd^	>1000 ^f^
15	19.1 ± 0.1 ^hi^	16 ± 3 ^i^	3.6 ± 0.1 ^gh^	6.9 ± 0.8 ^d^	1090 ± 52 ^bc^	>1000 ^f^
16	392 ± 6 ^c^	470 ± 3 ^c^	44 ± 1 ^c^	17 ± 1 ^bc^	/ ^g^	206 ± 3 ^a^
17	8.2 ± 0.6 ^i^	9.2 ± 1.6 ^j^	3.6 ± 0.1 ^gh^	0.7 ± 0.4 ^g^	3096 ± 26 ^de^	>1000 ^f^
KA					50 ± 14 ^a^	
EGCG						348 ± 9 ^b^

Different lowercase letters indicate statistically significant differences, determined by Tukey’s multiple comparison test for DPPH, ABTS, TPC, and TFC, and by Dunn’s multiple comparison test for TI and EI (*p* < 0.05).

**Table 3:** Docking energies and calculated dissociation constants (Kd) of the studied compounds for the Tyrosinase and Elastase enzymes.

Enzyme	Compound	Docking Energy, kcal/mol	Kd, μM
Tyrosinase	CAFFA	−4.14	916
	*p*-COUM	−4.38	611
Elastase	GA	−4.97	226
	AST	−6.52	16.4

## Data Availability

The original contributions presented in the study are included in the article/Supplementary Materials, further inquiries can be directed to the corresponding author.

## Supplementary Materials

The following supporting information can be downloaded at: https://www.mdpi.com/article/10.3390/plants14182869/s1. Table S1: List of HPTLC codes, common and Latin names of the investigated petal-derived plant taxa and their families, collection sites, and extraction yields (expressed as the mass of dried extract relative to the mass of dried petals, in %); Figure S1: Percentages (%) of HaCaT cell viability after treatment with the investigated PEs (100, 500, and 1000 µg/mL concentrations). Table S2. Calibration parameters of the phenolic standards, including calibration equations, correlation coefficients (*R*^2^), number of calibration curve points (N), and retention times (RT, min). Protocol S1. Quantification of phenolic compounds using UHPLC-DAD-MS.

## Abbreviations

The following abbreviations are used in this manuscript:

AES	Aesculetin
ABTS	2,2′-Azino-bis(3-ethylbenzothiazoline-6-sulfonic acid)
AST	Astragalin
CA	Chlorogenic acid
CAFFA	Caffeic acid
CTRL	Control
DMEM	Dulbecco’s Modified Eagle’s Medium
DMSO	Dimethyl sulfoxide
EC	Epicatechin
ECM	Extracellular matrix
EGCG	Epigallocatechin gallate
EI	Elastase inhibition
FET	Fish Embryo Toxicity
FBS	Fetal Bovine Serum
GA	Gallic acid
HaCaT	Human Immortalized Keratinocytes
Hpf	Hours post fertilization
HPTLC	High-performance thin-layer chromatography
IC50	Half maximal inhibitory concentration
ISOQ	Isoquercetin
ISORH	Isorhamnetin
ISORH-3-*O*-G	Isorhamnetin-3-O-glucoside
ISORH-3-*O*-R	Isorhamnetin-3-*O*-rutinoside
K	Kaempferol
L-DOPA	3,4-Dihydroxy-L-phenylalanine
LU	Luteolin
MTT	3-(4,5-Dimethylthiazol-2-yl)-2,5-diphenyltetrazolium bromide
NAR	Naringin
NEAA	Non-essential amino acids
NMR	Nuclear Magnetic Resonance
NPR	Natural Product Reagent
PEs	Petal extracts
PPE	Porcine pancreatic elastase
PEG	Polyethylene Glycol
Q	Quercetin
Q-3-*O*-G	Quercetin-3-*O*-rhamnoside
Q-3-*O*-R	Quercetin-3-*O*-glucoside
RS	Radical scavenging
RU	Rutin
TE	Trolox equivalents
TFC	Total Flavonoid Content
TI	Tyrosinase inhibition
TPC	Total phenolic content
UHPLC-	Ultra-High-Performance Liquid Chromatography coupled with Tandem
MS/MS	Mass Spectrometry
