# Supplementary material for: Multidisciplinary Bioanalytical Approach to Assess the Anti-Aging Properties of Flower Petals—A Promising Sustainable Cosmetic Ingredient
Source: Plants (Basel). 2025 Sep 15;14(18):2869. doi: 10.3390/plants14182869 (PMC12473510; doi:10.3390/plants14182869)
Supplement: Supplementary file 1 [file plants-14-02869-s001.zip › plants-3811765-supplementary.pdf]

Supplementary material

Multidisciplinary Bioanalytical Approach to Assess the Anti-Aging Properties of Flower Petals—A Promising Sustainable Cosmetic Ingredient

Đurđa Ivković [1], Milan Senćanski [2], Mirjana Novković [3], Jelena Stojković-Filipović [4], Jelena Trifković [5], Petar Ristivojević [5,*] and Maja Krstić Ristivojević [6,*]

[1] Innovative Centre of the Faculty of Chemistry Ltd., Studentski Trg 12-16, 11158 Belgrade, Serbia; djurdja@chem.bg.ac.rs
[2] Laboratory of Bioinformatics and Computational Chemistry, Institute of Nuclear Sciences Vinca,
National Institute of the Republic of Serbia, University of Belgrade, 11001 Belgrade, Serbia; sencanski@vin.bg.ac.rs
[3] Group for Muscle Cellular and Molecular Biology IMGGE, Institute of Molecular Genetics and Genetic Engineering, University of Belgrade, Vojvode Stepe 444a, 11042 Belgrade, Serbia;
mirjana.novkovic@imgge.bg.ac.rs
[4] Clinic of Dermatovenereology, University Clinical Center of Serbia, University of Belgrade-Faculty of
Medicine, Dr Subotića Starijeg 8, 11000 Belgrade, Serbia; j.stojkovic-filipovic@med.bg.ac.rs
[5] Department of Analytical Chemistry, University of Belgrade-Faculty of Chemistry, Studentski trg 12-16, 11158 Belgrade, Serbia; jvelicko@chem.bg.ac.rs
[6] Department of Biochemistry, University of Belgrade-Faculty of Chemistry, Studentski trg 12-16,
11158 Belgrade, Serbia
* Correspondence: ristivojevic@chem.bg.ac.rs (P.R.); krstic_maja@chem.bg.ac.rs (M.K.R.); Tel.: +381-11-333-66-71 (P.R.); +381-11-333-66-76 (M.K.R.)

List of figures:

Figure S1: Percentages (%) of HaCaT cell viability after treatment with the investigated PEs (100, 500, and 1000 μg/mL concentrations)

List of tables:

Table S1. List of HPTLC codes, common and Latin names of the investigated petal-derived plant taxa and their families, collection sites, and extraction yields (expressed as the mass of dried extract relative to the mass of dried petals, in %)

Table S2. Calibration parameters of the phenolic standards, including calibration equations, correlation coefficients (R^2), number of calibration curve points (N), and retention times (RT, min).

List of methods:

Protocol S1. Quantification of phenolic compounds using UHPLC-DAD-MS

Figure S1.

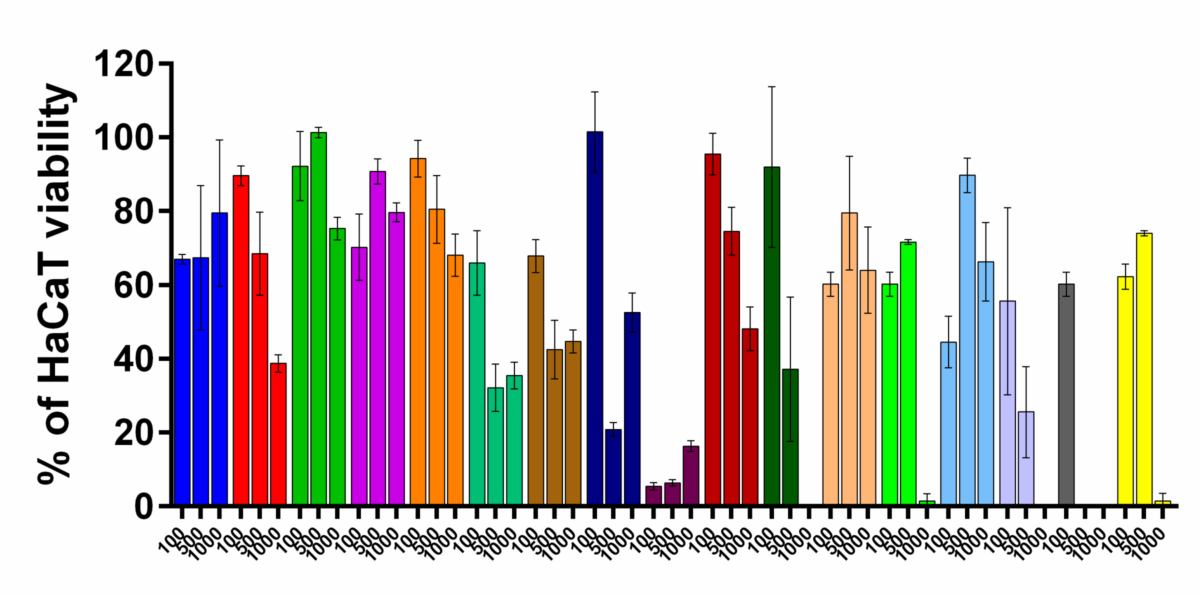

Table S1.

No.	Common name	Taxa Latin name	Family	Collection site	Extraction yield (%)
1	Prairie rose (purple)	*Rosa setigera Michx.*	Rosaceae	Lazarevac, Serbia	23.39%
2	Purple-yellow rose	*Rosa × odorata (Andrews) Sweet Valentina™*	Rosaceae	Lazarevac, Serbia	16.53%
3	White rose	*Rosa abietina Gren.ex Christ*	Rosaceae	Lazarevac, Serbia	10.96%
4	Damask rose, red	*Rosa × damascena* Mill.	Rosaceae	Lazarevac, Serbia	15.97%
5	Damask rose, yellow	*Rosa × damascena Herrm.*	Rosaceae	Lazarevac, Serbia	17.34%
6	Common white peony	*Paeonia officinalis* L.	Paeoniaceae	Lazarevac, Serbia	41.51%
7	Wild pink peony	*Paeonia mascula* (L.) Mill.	Paeoniaceae	Lazarevac, Serbia	32.58%
8	Horse chestnut	*Aesculus hippocastanum* L.	Sapindaceae	Lazarevac, Serbia	20.01%
9	Red horse-chestnut	*Aesculus x carnea Zeyx.*	Sapindaceae	Belgrade, Serbia	7.34%
10	Common lilac	*Syringa vulgaris*	Oleaceae	Lazarevac, Serbia	12.37%
11	Hoary mock-orange	*Philadelphus pubescens Loisel*	Hydrangeaceae	Lazarevac, Serbia	20.01%
12	Garden tulip	*Tulipa gesneriana* L.	Liliaceae	Lazarevac, Serbia	15.98%
13	Orange lily	*Lilium bulbiferum* L.	Liliaceae	Lazarevac, Serbia	2.43%
14	Wallflower	*Erysimum x cheiri (L.) Crantz*	Brassicaceae	Lazarevac, Serbia	19.58%
15	Pot marigold	*Calendula officinalis* L.	Asteraceae	Josif Pančić Pharmacy	16.06 %
16	Ivy geranium	*Pelargonium x peltatum* (L.) *L'Hér. ex Aiton*	Geraniaceae	Lazarevac, Serbia	10.45 %
17	Black locust	*Robinia pseudoacacia* L.	Fabaceae	Lazarevac, Serbia	10.86

Table S2.

Class	Compound	Calibration equation	R^2	N	RT (min)
Phenolic acid	GA	Y =-39972.3+878796x	0.9959	7	1.92
	CA	Y = -259774+8.12935e+006x	0.9920	7	4.92
	p-COUM	Y = 16511.2+669672x	0.9906	7	6.19
	CAFFA	Y = -157197+1.08067e+007x	0.9980	7	5.33
HQ	AES	Y = -102582+6.23295e+006x	0.9923	7	5.30
Flavonoid glycosides	RU	Y = -292667+8.60672e+006x	0.9968	7	6.03
	AST	Y = 61754.4+1.29988e+007x	0.9778	7	6.55
	HYP	Y = -53503.4+6.30816e+006x	0.9926	7	6.19
	NAR	Y = -17471.1+893596x	0.9950	7	6.37
	ISORH-3-*O*-R	Y = -17472.3 + 894126x	0.9950	7	6.45
	ISORH-3-*O*-G	Y = 1.77291e+006x	0.9778	7	6.63
	Q-3-*O*-R	Y = -77080.4+4.56381e+006x	0.9954	7	6.63
Flavonoids	EC	Y = -4488.72+374844x	0.9821	7	4.91-5.08
	Q	Y = -87980.8+2.38932e+006x	0.9966	7	7.93
	LU	Y = -87980.8+2.38932e+006x	0.9936	7	7.76
	K	Y = -43114.9+392203x	0.9917	7	8.79-8.80
	ISORH	Y = -145871+1.74866e+006x	0.9902	7	8.96

Protocol S1.

Phenolic compounds in the PEs were analyzed using a Dionex Ultimate 3000 UHPLC system (Thermo Fisher Scientific, Waltham, MA, USA) coupled to a diode array detector (DAD) and a triple quadrupole mass spectrometer (TSQ Quantum Access Max, Thermo Fisher Scientific). Separation was achieved on a Hypersil Gold C18 column (50 × 2.1 mm, 1.9 µm), with a mobile phase of 0.2% acetic acid in water and acetonitrile, at a flow rate of 0.4 mL/min and injection volume of 10 µL. The qqqMS operated with a HESI source in negative ion mode under the following conditions: spray voltage 4000 V, vaporizer 450 °C, capillary 320 °C, sheath gas 50 AU, auxiliary gas 20 AU, and collision energy 30 eV. Standard solutions were prepared from stock solutions (100 mg/mL) and diluted in methanol to 0.025–1.000 mg/L (X). Calibration curves ($R^2 > 0.9998$) were generated from peak areas (Y) versus concentration (X), and quantification was carried out using external calibration.
